# Optimizing the Mechanoluminescent Properties of CaZnOS:Tb via Microwave-Assisted Synthesis: A Comparative Study with Conventional Thermal Methods

**DOI:** 10.3390/ma16093511

**Published:** 2023-05-03

**Authors:** Franca C. Ugbo, Stefania Porcu, Riccardo Corpino, Andrea Pinna, Carlo Maria Carbonaro, Daniele Chiriu, Philippe F. Smet, Pier Carlo Ricci

**Affiliations:** 1 Department of Physics, University of Cagliari, S.p. no. 8 Km 0700, 09042 Monserrato, Cagliari, Italy; 2 LumiLab, Department of Solid State Sciences, Ghent University, Krijgslaan 281-S1, B-9000 Ghent, Belgium

**Keywords:** mechanoluminescence, microwave-assisted synthesis, phosphors, green synthesis

## Abstract

Recent developments in lighting and display technologies have led to an increased focus on materials and phosphors with high efficiency, chemical stability, and eco-friendliness. Mechanoluminescence (ML) is a promising technology for new lighting devices, specifically in pressure sensors and displays. CaZnOS has been identified as an efficient ML material, with potential applications as a stress sensor. This study focuses on optimizing the mechanoluminescent properties of CaZnOS:Tb through microwave-assisted synthesis. We successfully synthesized CaZnOS doped with Tb3+ using this method and compared it with samples obtained through conventional solid-state methods. We analyzed the material’s characteristics using various techniques to investigate their structural, morphological, and optical properties. We then studied the material’s mechanoluminescent properties through single impacts with varying energies. Our results show that materials synthesized through microwave methods exhibit similar optical and, primarily, mechanoluminescent properties, making them suitable for use in photonics applications. The comparison of the microwave and conventional solid-state synthesis methods highlights the potential of microwave-assisted methods to optimize the properties of mechanoluminescent materials for practical applications.

## 1. Introduction

Nowadays, there is an extensive demand for innovative materials and techniques that allow us to reduce energy consumption and guarantee the sustainable development of different fields, such wind turbines, solar panels, electric beams, energy-efficient lighting (LEDs), consumer electronics, durable metals, automotive, aerospace, and medical equipment.

In the field of chemistry, the use of mechanical energy has already been widely utilized for providing a solution-free, energy-saving, high-productivity, and low-temperature process [1,2,3]. In the field of phosphors and sensors, the mechanoluminescent effect is increasing its importance due to the development of new intriguing materials [4,5]. Inner-crack visualization, biological stress, light source, and ultrasonic powder recording are just few already developed fields of application [6,7,8]. Mechanoluminescent materials can be activated not only through impacts, but also through sound waves, loads, and, indirectly, through the application of external magnetic fields [9,10,11,12].

A key element was the development of materials with high efficiency in the transformation of the mechanical stress in optical emission. Among these, a specific place is held by CaZnOS whose mechanoluminescent properties were published for the first time only in 2013 and its first structural characterizations just 8 years earlier [13]. CaZnOS, doped (and co-doped) with rare earth elements such as Eu, Tb, Er, Pr, Sm, Er, and Tm, or with transition metals (Cu, Al, or Mn), is highly efficient both as a phosphor and as a mechanoluminescent material depending on the structural defects of the matrix and how the emitter element interacts with the defects themselves [14,15,16,17,18,19,20]. The structure can be easily doped with rare earth elements for the similar ionic radius of Ca, and, among the other Tb, represents one of the popular dopants of high green luminescence from its ^5^D_4_−^7^F_J_ transition (where J = 3, 4, 5, and 6), with a recombination time spanning from microseconds to milliseconds (ms-ms). Further, Tb^3+^ is usually co-doped with other rare earth elements in a different host matrix, acting both as a sensitizer and activator [15,21,22].

In the CaZnOS structure, the shallow traps connected to the Ca vacancies are responsible for the high mechanoluminescence effects, as well as for the persistent luminescence. Actually, when a mechanical load is applied, a local piezoelectric field is produced, causing band bending and triggering the release of trapped charges to the recombination sites (i.e., the dopants) [23].

Therefore, defects play a crucial role in obtaining a well-defined crystalline structure, which can be achieved through easily reproducible synthesis processes. The traditional method for obtaining CaZnOS involves a solid-state synthesis process that is conducted at high temperatures between 1050 and 1200 °C for a period of 4–8 h. This method is known for the excellent reproducibility of the resulting materials, but it is also energy-intensive. The heating process is slow and complex, requiring a long processing time to convert the materials into useful products. Additionally, the thermal energy transfer from the source is often inefficient, as it must pass through the walls of the container before reaching the substance inside, leading to the insufficient heating of the material.

To overcome the time-consuming process and achieve a material with a highly efficient luminescence, researchers and scientists are exploring the use of microwave technology for synthesizing phosphor materials [24,25].

The use of microwave (MW) methods for the synthesis of materials offers several advantages compared to other methods such as sol-gel, solid-state, combustion-assisted, and hydrothermal synthesis. One of the main advantages of MW methods is the drastic reduction in synthesis time, making it a fast and efficient method. Additionally, the equipment required for MW synthesis is relatively simple and easy to use, requiring no specialized knowledge or skills to operate. This also makes it a cost-effective method as it does not require expensive equipment or specialized facilities.

MW methods have been found to produce high-quality materials with a high yield, making them ideal for industrial applications. Additionally, the materials produced using MW methods are eco-friendly as they consume less energy and produce less waste than traditional methods [24]. The powder materials produced by MW methods are also suitable for industrial applications without the need for annealing to improve their optical properties. Overall, MW methods offer a flexible and cost-effective method for the synthesis of new materials in the form of crystals or liquids, with improved properties that are readily applicable for industrial use Inizio modulo.

The rapid and uniform heating of the reaction mixture is attributed to the dielectric properties of the materials, which enable the absorption of microwave energy and subsequent conversion into heat. The interaction between microwaves and the reaction mixture is dependent on the frequency of the radiation, the dielectric properties of the materials, and the geometry of the reaction vessel. As the microwaves penetrate the reaction mixture, they induce a rotational and vibrational motion of the molecules, leading to a rapid increase in temperature. The localized and rapid heating of the reaction mixture can facilitate the formation of the desired products, resulting in shorter reaction times and higher yields. This is due to the rapid and localized heating of the reaction mixture, which can lead to the formation of nucleation sites and the subsequent growth of crystals in a controlled manner.

Overall, the use of microwave-assisted synthesis offers several advantages over conventional methods, including shorter reaction times, higher yields, and the formation of unique crystal structures and morphologies.

**Figure 1:**
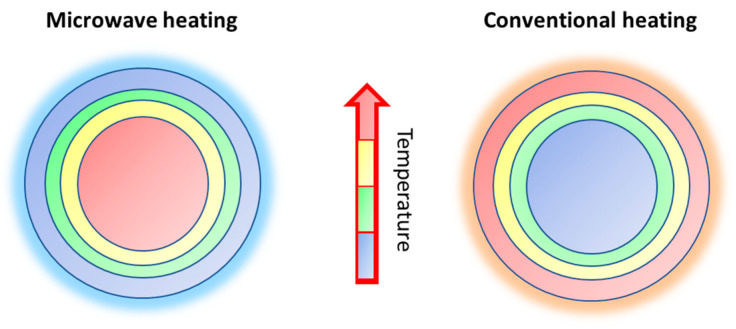
Schematic representation of microwave heating vs. conventional heating.

As sketched in the scheme of Figure 1, microwave irradiation generates heat within the material and throughout its entire mass simultaneously, as this excitation process results in a consistent and uniform heating of the material, leading to a rapid and homogenous increase in temperature.

Often, the use of MW techniques is assisted by a thermal effect with the scope to start the real MW reaction (generating an intermediate compound) or to assist the synthesis by increasing the ion mobility [2].

Microwave-assisted synthesis (MAS) is a relatively new method for the synthesis of a wide range of materials, including both pure and doped organic, inorganic, metallic, ceramic, polymer, composite, and sulfide phosphors. It is a fast and efficient method that can significantly reduce the synthesis time compared to traditional methods. The technique is based on the use of microwave energy to heat and excite the molecules of the material being synthesized, leading to a rapid and homogenous increase in temperature, and has many advantages over traditional synthesis methods, including:

- Reduced synthesis time;
- Consistent and uniform heating of the material;
- High-quality materials with high yield;
- Eco-friendly, as it consumes less energy and produces less waste than traditional methods;
- Cost-effective, as it requires less equipment and facilities than traditional methods.

Furthermore, it is a versatile technique that can be used to synthesize a wide range of materials with improved properties and are readily applicable for industrial use [26,27].

MAS has proven to be a practical method for synthesizing both pure and doped organic, inorganic, metallic, ceramic, polymer, composite, and sulfide phosphors [14,15], such as Y_2_O_3_:Eu^3+^ [28], CaCO_3_:Eu^3+^ [29], CaGd_2_(WO_4_)_4_: Er^3+^/Yb^3+^ [30], and Gd_2_O_2_S: Tb^3+^ [31].

It is worth noting that several studies have demonstrated the effectiveness of MAS in synthesizing various inorganic phosphor materials, with excellent results. For example, Carvalho et al. [32] successfully used microwave methods to synthesize high-quality persistent luminescence materials, such as Sr_2_MgSi_2_O_7_:Eu^2+^ and Dy^3+^. In another study conducted by Jiawei Bi et al. [28], Eu^3+^-doped Y_2_O_3_ nanophosphors were synthesized using a simple, ultra-fast, and environmentally friendly method combining ultrasonic and microwave techniques, obtaining a material with excellent optical properties.

In the present study, samples of CaZnOS doped with Tb were synthesized using the MAS technique, with synthesis times of 10, 20, and 30 min. The samples were characterized using a multi-technique approach with particular attention to the mechanoluminescence properties. Afterwards, the structural, morphological, and optical properties were compared to those of the sample obtained via solid-state synthesis.

The results of this study demonstrated that the microwave-assisted synthesis method is a valid and cost-effective method for the synthesis of phosphors with enhanced mechanoluminescence efficiency when compared to the sample obtained using solid-state synthesis. Overall, this study highlights the potential of microwave-assisted synthesis as a promising method for the production of efficient and cost-effective oxysulfide phosphors.

## 2. Experimental

### 2.1. Materials and Methods

Terbium doped CaZnOS samples (5 wt%) were prepared by MAS method starting from CaCO_3_, ZnS, Tb_4_O_7_, and Li_2_CO_3_ powders:

(1 − 0.05)CaCO_3_ + ZnS + 0.0125Tb_4_O_7_ → Ca_(0.95)_ZnOSTb_0.05_ + CO_2_

Due to the similar ionic radius (99 pm for Ca^2+^, 74 pm for Zn^2+^ 4-co-ordinate, and 95 pm for Tb^3+^ in 6-coordination), Tb^3+^ ions substitute Ca^2+^ ions. However, considering charge states larger than Ca^2+^, Li_2_CO_3_ was necessary for charge balance purposes and as reaction catalyst. It was added in excess with respect to the stoichiometry amounts both in the solid-state and microwave-assisted synthesis. To obtain about 0.8 g of Tb-doped CaZnOS, the following amounts were utilized: 0.4795 g of CaCO_3_, 0.4870 g of ZnS, and 0.0045 g Tb_4_O_7_.

**Figure 2:**
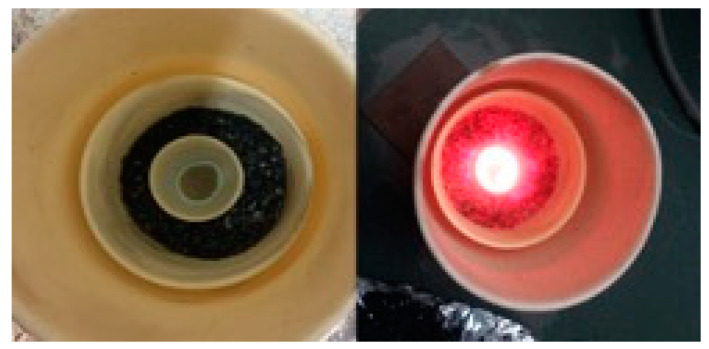
View of the microwave-assisted synthesis (MAS). On the (**left**): the sample after the synthesis; on the (**right**): during the synthesis.

MAS synthesis: The mixture was transferred to a small crucible (10 mL), which was placed within a larger crucible containing activated charcoal. The larger crucible was covered with a lid and placed in the microwave with a power of 750 W. The samples were microwaved for 10, 20, and 30 min, respectively, and then left to cool down naturally in the air (hereafter referred to as MW10 min, MW20 min, and MW30 min, respectively). Figure 2 reports the photographs of the sample during the synthesis (the thermal effect is clearly visible) and after it was cooled down to room temperature. It is worth noting that a thin carbon layer is deposited on the top surface of the sample. This layer has been removed from the samples before the study, even if a small percentage of carbon cannot be totally excluded.

Additional samples were prepared without the use of activated charcoal to investigate the effect of the charcoal as an additional heat absorber on the synthesized materials.

Solid-state (SS) synthesis: The precursor mixture was placed in an alumina crucible (10 mL) and then sintered in a tubular furnace under a constant flow of nitrogen gas. The temperature was gradually increased to 1000 °C at a ramp rate of 10 °C/min and held at that temperature for 5 h. After that, the samples were allowed to cool down naturally to room temperature (hereafter named SS sample).

### 2.2. Characterization Techniques

The powders were characterized using X-ray diffraction, PL, Raman, mechanoluminescence, and time-resolved luminescence to check the structural, optical, and ML properties.

Raman spectroscopy measurements were performed using an MS750 spectrograph (Sol-Instruments) equipped with a 600 gr/mm grating. A 785 nm laser was used as the excitation source, focused through a 10 × Olympus objective lens with an estimated laser power of 7.5 × 10^3^ W/cm^2^. No heating effect on the samples has been revealed. The measurements were conducted at room temperature, with a spectral resolution of 1 cm^−1^.

X-ray diffraction measurements were conducted using a Bruker D8 Advance diffractometer operating at 30 kV and 20 mA. The diffractometer was equipped with a Cu tube as the X-ray source (wavelength of 1.5418 Å) and a Vantec-1 PSD detector. The powder patterns were collected in the 2θ range of 10° to 70°.

Steady-state photoluminescence measurements were conducted using a filtered light from a laser-driven Xenon lamp (EQ-99X) that had a final bandwidth of approximately 1 nm. The emitted photoluminescence was collected using an optical fiber and directed to an Avantes Thermo-Electric Cooled spectrometer for analysis.

Time-resolved luminescence measurements were performed using an optical parametric oscillator with a frequency doubler device, which was pumped by the third harmonic (355 nm) of a pulsed Nd:YAG laser (Quanta Ray Pro 730). The excitation pulse width at half-maximum was 8 ns with a repetition rate of 10 Hz, and a spectral bandwidth less than 0.3 cm^−1^. The collected signal was dispersed by a spectrograph (Arc-SpectraPro 300i) with a spectral bandpass of less than 2.5 nm and detected by a gateable intensified CCD (PI MAX Princeton Inst.). To minimize the dark current, the detector was cooled to −20 °C using a Peltier device.

### 2.3. Mechanoluminescence Test

The mechanical pulse for the mechano-emission was generated by dropping a stainless-steel ball (10 mm diameter and 8.15 g) from a height of about 1 m onto layers of powder approximately 0.5 mm thick. The impact speed was measured to be 4 m/s using an Airsoft E9800-X anemometer. The impact zone was placed on a transparent CaF_2_ substrate, and the emitted light was collected from the opposite side and focused onto an intensified CCD (PI MAX Princeton Inst.).

## 3. Results and Discussion

Structural characterization plays a crucial role in this study, as it provides insight into the crystal structure and phase purity of the samples.

**Figure 3:**
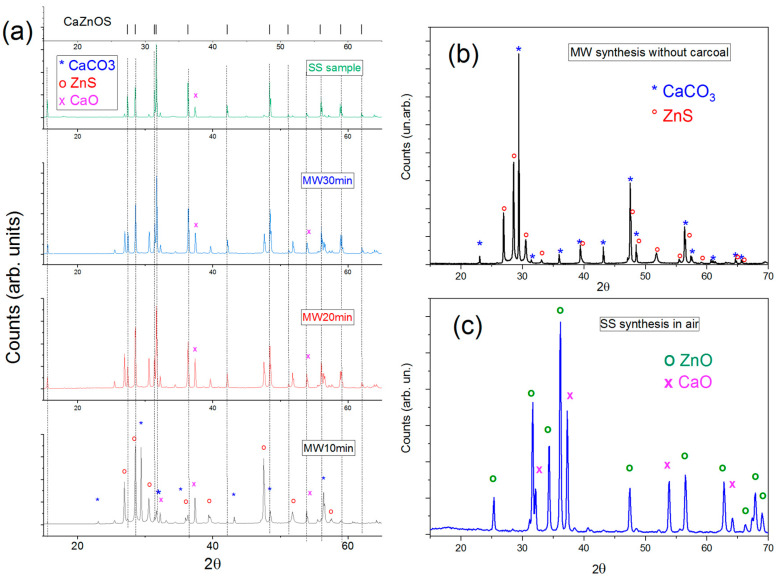
(**a**) X-ray diffraction patterns of SS- and MW-synthesized Tb-doped CaZnOS samples. The vertical dashed lines indicate the diffraction angles for CaZnOS. The main diffraction lines for impurity phases are indicated as well. (**b**) X-ray diffraction pattern of microwave-synthetized sample in absence of Caracoal, (**c**) X-ray diffraction pattern of solid-state sample growth in air.

Figure 3 displays the X-ray diffraction patterns of samples synthesized using the MAS approach at different time intervals. Additionally, for comparison, the XRD pattern of the sample obtained through the SS reaction method is also included in the figure to provide a clear comparison of the structural properties of the samples synthesized using the different techniques.

The SS sample pattern, analyzed by means of the software MAUD for multi-pattern fitting [33], reveals the presence of CaZnOS (Reference ICSD card no. 245309) with a percentage of 90% and residuals of ZnS and CaO in a low percentage.

In the XRD patterns of the MAS samples, the presence of the starting precursors is clearly visible. As expected, the sample obtained at the shortest irradiation time has a higher content of unreacted precursors, which decreases with the duration of the microwave treatment. The CaZnOS phase is observed to increase from around 35% after 10 min, with a high percentage of precursors CaCO_3_, CaO, and ZnS, to 60% at 20 min, where residuals of CaO and ZnS are also present in moderate percentages. Further increases in the synthesis time do not result in a significant increase in the weight percentage of CaZnOS, nor in the formation of any other competitive reactions.

The presence of CaO can be strictly related to the chamber atmosphere. It was already observed that a reducing atmosphere is strictly required for the formation of CaZnOS, while the growth in air generates only oxides. In the MAS process, the reducing atmosphere is provided by the high percentage of CO generated by the thermal effect of the charcoal present in the outer crucible [20]. In the absence of charcoal, the CaZnOS structure was not formed, both for the presence of a not-reducing atmosphere (in the SS synthesis in air, only CaO and ZnS are formed) and also for the lower temperature of the starting precursor (Figure 3b,c).

**Figure 4:**
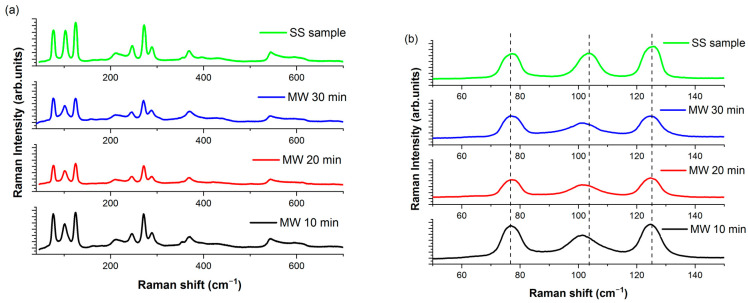
(**a**) Raman spectra of SS- and MW-synthesized Tb-doped CaZnOS samples. (**b**) Enlarged view of the 50–150 cm^−1^ region.

The Raman spectrum of the SS-CaZnOS sample displays all the characteristic bands expected in the CaZnOS structure (Figure 4a), with the most prominent ones at 75 cm^−1^, 102 cm^−1^, 124 cm^−1^, and 272 cm^−1^ [20].

These peaks correspond to the double degenerate vibration E modes, specifically the anti-symmetric E_2_ mode and the symmetric E_1_ mode. The region at lower wavenumbers (50 cm^−1^–150 cm^−1^) is sensitive to the vibrations of heavier elements and can provide information about the presence of rare earth dopant elements in the sample [15].

The findings from the Raman measurements are consistent with the results obtained from the X-ray diffraction analysis.

The Raman spectrum of the MW10min sample displays the main characteristics of the CaZnOS matrix; however, an additional peak at 348 cm^−1^ is also observed, providing evidence of the presence of ZnS in the sample.

However, a general comparison of the Raman spectra shows a similar behavior across all samples, with the main variations being observed only in the low-energy region (50–150 cm^−1^).

Three main bands are clearly visible in the Raman spectra of the samples (Figure 4b), corresponding to the two E2 modes at 75 and 102 cm^−1^ and the E1 mode at 125 cm^−1^. It is noteworthy that these bands remain centered at the same Raman frequency and show no significant variations in their width, as can be seen when comparing the spectra of the samples grown by MAS and the SS synthesis method.

On the contrary, a clear variation can be observed in the central E2 band at 102 cm^−1^, both in position (the band is peaked at 100 cm^−1^ in the MW samples) and band width. This band is primarily associated with the vibrations of the Ca ions in the lattice, while the other two modes are primarily related to the vibrations of the Zn ions in the lattice [15].

The presence of impurity phases (such as CaO) in the samples may introduce lattice defects associated with Ca and O vacancies in the CaZnOS matrix. These defects can result in a broadening and shifting of the Raman bands due to the altered short-range order near the Ca vacancies.

**Figure 5:**
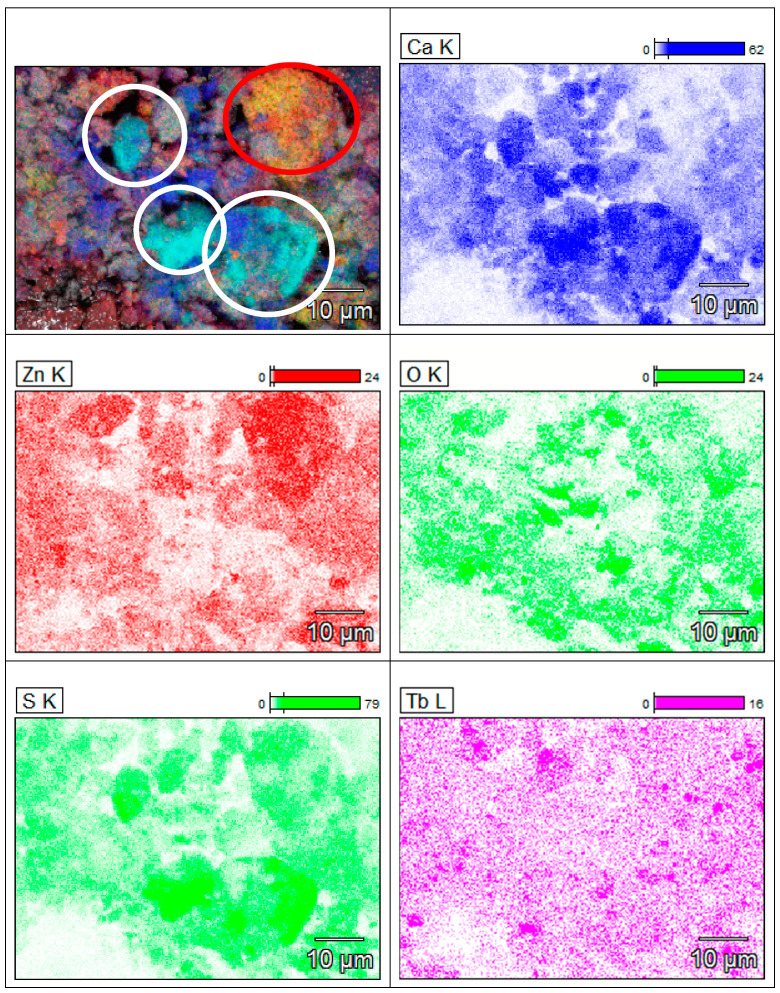
Results of EDAX analysis of the MW20 min sample. The white and red circles indicate the presence of clusters of calcium and zinc, respectively.

The elemental distribution obtained from the EDAX measurements can give an important indication and proof of local inhomogeneities. Even if the CaZnOS structure is the predominant phase, a deep analysis permits us to find some aggregates of different structures. Figure 5 shows the presence of inhomogeneous zones with clusters of Ca and S (the most probable is CaS—white circle), and Zn and S (ZnS—red circle). However, the presence of CaS was relatively low (under the detection limit of the XRD results) and the inhomogeneities are restricted to a limited number of zones.

**Figure 6:**
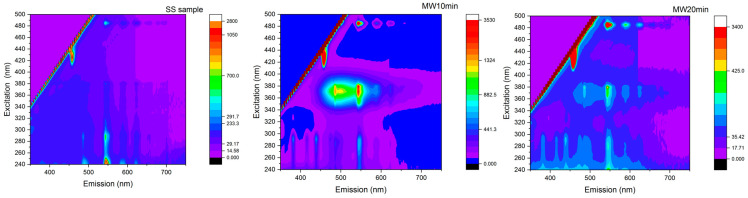
3D-plot of the excitation and emission spectra for SS samples, MW10 min, and MW20 min samples.

The optical properties confirm the similarity between the sample obtained by solid-state synthesis and the MAS growth sample (MW20 min) (Figure 6).

The main features of the photoluminescence spectra come from the recombination at the Tb sites, where the narrow emissions below 500 nm are related to the transitions from the ^5^D_3_ excited levels to the ^7^F_J_ ground states of the rare earth, while the main features in the 490–600 nm spectral region are due to the recombination from the ^5^D_4_ levels [34].

The excitation spectrum of the SS sample presents a direct excitation channel in the deep UV region (240–280 nm), related to the band-to-band excitation of the matrix and direct excitation of the Tb levels (Figure 6).

The MAS sample at 10 min presents a broad emission band centered at about 500 nm with the excitation between 340 and 400 nm. Upon increasing the duration of the microwave treatment, this band decreases its importance. As was already indicated in previous works [20,35,36], the latter band is due to the presence of oxygen vacancies, VO^2+^, generated as consequences of the Ca and S vacancies, and can be efficiently utilized as a sensitizer for the emission at the rare-earth elements site. Further and dedicated measurements should be addressed to fully confirm this assignment.

**Figure 7:**
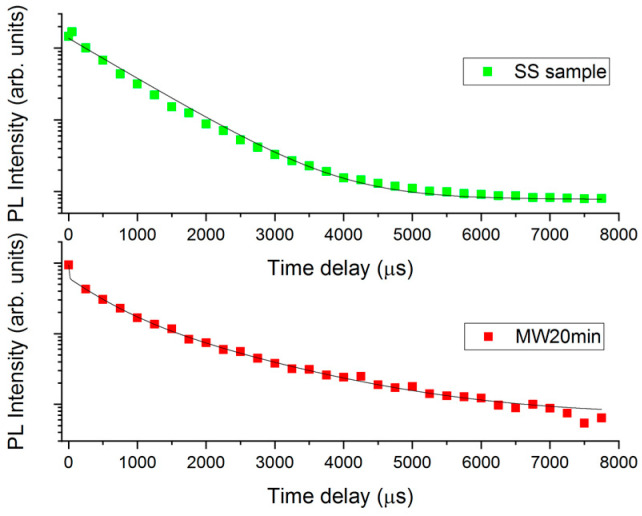
Time-decay behavior of SS and MW20 min samples.

Time-resolved measurements provide a deeper understanding of the different distributions in the samples obtained through the SS and MAS methods (Figure 7).

In the SS sample, the decay from the ^5^D_4_ to ^7^F_5_ level is monitored at 545 nm with the excitation at 280 nm, and follows a single exponential behavior with a time constant of about 900 microseconds. This value suggests the presence of non-radiative recombination pathways [34]. Further, the single exponential behavior suggests that the decay is dominated by only one type of recombination path in the SS sample.

On the other hand, the recombination in the MW sample exhibits a more complex behavior due to the presence of various non-radiative recombination pathways. It is possible to use the following equation to obtain a quantitative value and determine an average lifetime: [37]

$$\tau =\frac{\int_{0}^{T}I\left(t\right)\cdot tdt}{\int_{0}^{T}I\left(t\right)dt}$$

where *I(t)* is the time-dependent photoluminescence intensity and *T* is the duration of the time window (8 ms in our case, or can be considered infinite in a more general case). The value of the average lifetime for the MW20 min and MW30 min samples is 769 μs and 750 μs, respectively.

**Figure 8:**
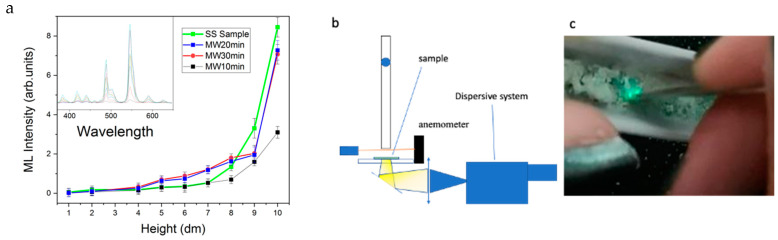
(**a**) Spectral shape of the light emitted (inset) and overall intensity from the peak at 545 nm of the Tb emission as function of the drop height, (**b**) schematic representation of the experimental set-up, and (**c**) image of the bright green emission by simple scratching of the synthesized powder in an alumina crucible.

Both the SS and MW20 samples exhibit strong mechanoluminescent properties, as evidenced by the intense light emitted during the mechanical pulse (Figure 8c). The green color of the emission confirms that the emitting centers are Tb ions, as further confirmed by the spectral dependence (inset in Figure 8a).

To more deeply define the behavior of the mechanoluminescent properties of the samples, the single impact effect has been studied. The samples were hit with a stainless-steel ball at different speeds, and the emitted light was collected through a CaF_2_ substrate. The powder layer of the samples, about 0.5 mm thick, was placed on the substrate to measure the light emitted upon impact (Figure 8b). However, it is important to note that not all the kinetic energy from the ball is transferred to the powder, as the ball also rebounds. This means that only a fraction of the impact energy is transferred to the samples.

From the height reached from the ball after the impact, it is possible to estimate the energy not transferred to the sample during the impact (about 20% for all the impacts). The inset in Figure 8a reports the spectral shape of the light emitted and the overall intensity from the peak at 545 nm of the Tb emission. The data reported in the main panel are the result of the average of five impacts.

As expected, the sample obtained with the synthesis time of 10 min produces a lower light intensity from the impacts. The samples synthesized using the MAS method at 20 and 30 min show a slightly lower intensity of mechanoluminescence compared to the SS sample, with a reduction of about 15% for the highest energy impact. At intermediate drop heights (between 0.4 and 0.7 m), the ML emission intensity for the MW10 and MW20-samples is well above the intensity for the SS samples, suggesting a lower threshold energy for ML emission.

## 4. Conclusions

In summary, this study demonstrated the potential of CaZnOS:Tb as a high-efficiency, chemically stable, and eco-friendly mechanoluminescent material for use in modern lighting and display technologies. Through the use of various analytical techniques, it was determined that the materials synthesized using MAS had similar structural, morphological, and optical properties to those synthesized using conventional SS methods, but with enhanced mechanoluminescent properties.

While the final structure of the MAS samples may contain a higher percentage of impurity compounds due to the absence of a strongly reducing atmosphere, the comparable efficiency of its emission upon single impacts suggests that microwave synthesis can still be a viable and advantageous method for certain applications. For example, MAS can offer faster reaction times, higher reaction yields, and greater control over reaction conditions, making it an attractive alternative to traditional SS synthesis. Additionally, MAS can be a more sustainable and energy-efficient process, with reduced solvent and energy consumption compared to other methods. Therefore, despite the presence of impurities, the efficiency of MAS samples indicates that this method has the potential for efficient and sustainable synthesis in various fields and for practical applications; however, more focalized efforts on the specific performance needs to be performed for a full optimization of the synthesis conditions.
